# Vivisection: An Appeal to Reason

**Published:** 1892-11-05

**Authors:** Lawson Tait


### Vivisection: An Appeal to Reason.

By Mr. Lawson Tait, F.R.C.S.

As you refer to me by name in your leading article on this subject, and as you misrepresent my views and my actions, no doubt unintentionally, but to some degree of importance, perhaps you will admit a short reply from me to your columns. I am quite of your opinion that most of the Englishmen who make experiments upon living animals are men of high character, of worthy motives, and of truly humane feeling. I have always done my best to persuade the "anti-vivisection part" that this is the case; and I deeply regret that it is necessary further to agree with you in the assumption that there is a single exception to this statement that we agree upon, for it is no business of mine to besmirch my profession. You say that it is inexplicable how a man of my knowledge and experience could have thought of proposing such a discussion in the daily newspapers. This requires a direct contradiction, for I did no such thing. The discussion as proposed at the Church Congress was not proposed by me, nor had I any part, direct or indirect, in the proposal. I was told that Sir Andrew Clark was to give an address in support of vivisection, and I was asked to reply to him. I agreed to do this, and then I was ruled out by the authorities of the Congress as not being a member of the Church of England, and, therefore, according to their rules, incompetent to address them. Then Sir Andrew Clark, Sir James Paget, Sir George Humphrey, and Dr. Wilkes gave vent to their opinions in the daily papers. They began the discussion, and I challenged them to continue it, and I should have permitted them to select their own organs or their own audience, provided only I got the same hearing they got and fair play. Let me say, in conclusion of this brief letter, that I do not take up the ethical or the religious ground. I take a purely material ground, to the effect that the method of research by experiments on animals is so fallacious, so entirely unscientific, and has been so dangerously misleading, that I wish to see it discontinued. Into Sir Andrew Clark's metaphysico-theological arguments concerning "vicarious sacrifice being a law of the physical, mental, moral, and spiritual life" I refuse to go, for two reasons. The first is that these theological grounds are precisely the grounds on which a vast number of people object to vivisection, and they must have a much deeper insight into such questions than Sir Andrew possibly can have, for a large number of them are professional theologians, whilst Sir Andrew is but an amateur. Secondly, I gave up metaphysics and theology long ago, and cannot argue on either. But on the uselessness of vivisection I can argue, and if you will permit me I shall do so, only not with anyone who uses bad language.

